# Supplementary material for: Identification of photoperiod-induced specific miRNAs in the adrenal glands of Sunite sheep (Ovis aries)
Source: Front Vet Sci. 2022 Jul 22;9:888207. doi: 10.3389/fvets.2022.888207 (PMC9354845; doi:10.3389/fvets.2022.888207)
Supplement: Supplementary file 1 [file Data_Sheet_1.ZIP › Supplementary file/miRDeep2 file.zip!/miRDeep2 file/Know_Structure/SP42A1/oar-mir-409.pdf]

miRBase precursor : oar-mir-409
Total read count : 11549
oar-miR-409-5p read count : 2295
oar-miR-409-3p read count : 9252
remaining reads : 2

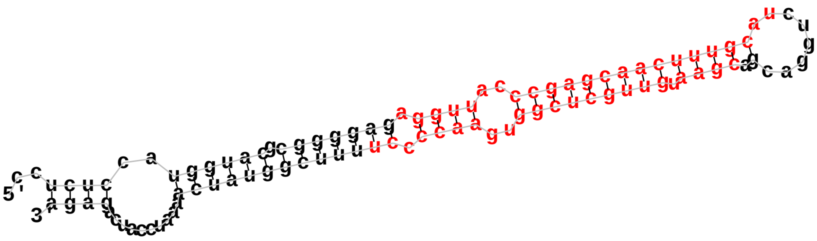

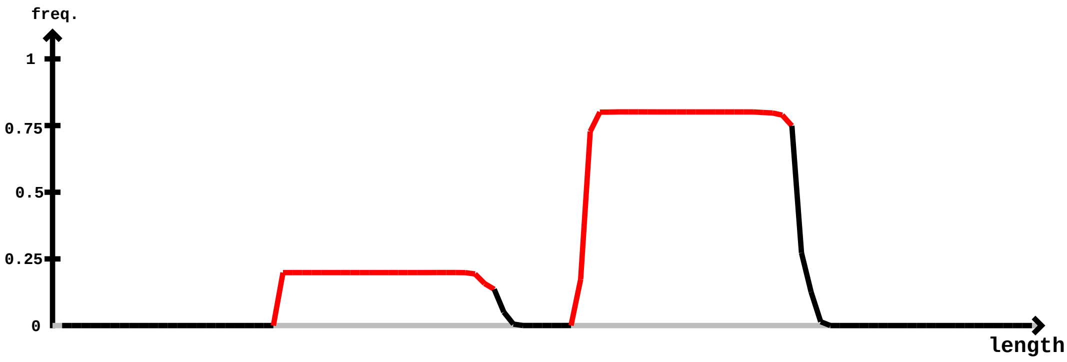


```
                      oar-miR-409-5p
                                                     oar-miR-409-3p
5'- ccucuccaugguacgcggggagagguuacccgagcaacuuugcaucuggacgacgaauguugcucggugaaccccuuuucgguaucaaaauccaucagaga  -3'   exp
    .((((...((((((.(((((((.((((..((((((((((((((........).)))).)))))))))..)))).))))))))))))).........)))).    reads    mm    sample
    ......................agguuacccgagcaacuuu............................................................    6        0     seq
    ......................agguuacccgagcaacuuug...........................................................    56       0     seq
    ......................agguuacccgagcaacuuugc..........................................................    416      0     seq
    ......................agguuacccgagcaacuuugca.........................................................    247      0     seq
    ......................agguuacccgagcaacuuugcau........................................................    988      0     seq
    ......................agguuacccgagcaacuuugcauc.......................................................    519      0     seq
    ......................agguuacccgagcaacuuugcaucu......................................................    62       0     seq
    .......................gguuacccgagcaacuuugcau........................................................    1        0     seq
    ......................................uuugcaucuggacgacgaaugu.........................................    1        0     seq
    ......................................uuugcaucuggacgacgaauguu........................................    1        0     seq
    ....................................................acgaauguugcucggugaac.............................    1        0     seq
    .....................................................cgaauguugcucggugaac.............................    20       0     seq
    .....................................................cgaauguugcucggugaacc............................    14       0     seq
    .....................................................cgaauguugcucggugaaccc...........................    32       0     seq
    .....................................................cgaauguugcucggugaacccc..........................    174      0     seq
    .....................................................cgaauguugcucggugaaccccu.........................    1282     0     seq
    .....................................................cgaauguugcucggugaaccccuu........................    338      0     seq
    .....................................................cgaauguugcucggugaaccccuuu.......................    113      0     seq
    .....................................................cgaauguugcucggugaaccccuuuu......................    21       0     seq
    .....................................................cgaauguugcucggugaaccccuuuuc.....................    1        0     seq
    ......................................................gaauguugcucggugaa..............................    1        0     seq
    ......................................................gaauguugcucggugaac.............................    2        0     seq
    ......................................................gaauguugcucggugaacc............................    8        0     seq
    ......................................................gaauguugcucggugaaccc...........................    52       0     seq
    ......................................................gaauguugcucggugaacccc..........................    261      0     seq
    ......................................................gaauguugcucggugaaccccu.........................    3656     0     seq
    ......................................................gaauguugcucggugaaccccuu........................    1229     0     seq
    ......................................................gaauguugcucggugaaccccuuu.......................    1069     0     seq
    ......................................................gaauguugcucggugaaccccuuuu......................    131      0     seq
    .......................................................aauguugcucggugaaccc...........................    9        0     seq
    .......................................................aauguugcucggugaacccc..........................    27       0     seq
    .......................................................aauguugcucggugaaccccu.........................    564      0     seq
    .......................................................aauguugcucggugaaccccuu........................    126      0     seq
    .......................................................aauguugcucggugaaccccuuu.......................    101      0     seq
```

```
                      oar-miR-409-5p
                                                     oar-miR-409-3p
ccucuccaugguacgcggggagagguuacccgagcaacuuugcaucuggacgacgaauguugcucggugaaccccuuuucgguaucaaaauccaucagaga

.......................................................aauguugcucggugaaccccuuuu......................   10   0   seq
........................................................auguugcucggugaaccccuu........................   1    0   seq
.........................................................uguugcucggugaaccccuu........................   1    0   seq
.........................................................uguugcucggugaaccccuuu.......................   5    0   seq
.........................................................uguugcucggugaaccccuuuu......................   3    0   seq
```
